# Spatiotemporal Trends and Drivers of PM_2.5_ Concentrations in Shandong Province from 2014 to 2023 Under Socioeconomic Transition

**DOI:** 10.3390/toxics13110978

**Published:** 2025-11-13

**Authors:** Shuaisen Qiao, Qingchun Guo, Zhenfang He, Genyue Feng, Zhaosheng Wang, Xinzhou Li

**Affiliations:** 1 School of Geography and Environment, Liaocheng University, Liaocheng 252000, China; 13863531736@163.com (S.Q.); hezhenfang@lcu.edu.cn (Z.H.); yue13847861122@163.com (G.F.); 2 Institute of Huanghe Studies, Liaocheng University, Liaocheng 252000, China; 3 State Key Laboratory of Loess Science, Institute of Earth Environment, Chinese Academy of Sciences, Xi’an 710061, China; lixz@ieecas.cn; 4 Key Laboratory of Atmospheric Chemistry, China Meteorological Administration, Beijing 100081, China; 5 National Ecosystem Science Data Center, Key Laboratory of Ecosystem Network Observation and Modeling, Institute of Geographic Sciences and Natural Resources Research, Chinese Academy of Sciences, Beijing 100101, China; wangzs@igsnrr.ac.cn

**Keywords:** PM_2.5_, socioeconomic factors, meteorological factors, geodetector, air pollution, air temperature, wind speed, emissions

## Abstract

China’s rapid economic growth since its reform and opening-up has come at the cost of worsening atmospheric pollution. This study investigates the spatiotemporal evolution and driving mechanisms of PM_2.5_ concentrations in Shandong province, a key industrial region, during 2014–2023, using comprehensive air quality monitoring, meteorological observations, and socioeconomic datasets. Through spatial analysis and geodetector methods, we identify that (1) The annual PM_2.5_ concentration decreases significantly by 50.9%; spatially, heterogeneity is observed with the western urban agglomeration experiencing more severe pollution, while the eastern coastal urban agglomeration exhibits better air quality. (2) Gravity model analysis shows that the centroids of PM_2.5_ pollution undergo distinct migration phases. (3) PM_2.5_ levels show a distinct seasonal pattern, peaking in winter at a level 143.7% higher than the summer average. (4) The meteorological driving factors are primarily air temperature (r = 0.511) and wind speed (r = −0.487), while the socioeconomic factors are tertiary industry production (r = −0.971), particulate matter emissions (r = 0.956), and sulfur dioxide emissions (r = 0.938). Concurrently, the combined effect of tertiary industry production and PM emissions account for 99.5% of PM_2.5_ variability. Notably, we validate an Environmental Kuznets Curve relationship (R^2^ = 0.805) between economic development and air quality improvement, demonstrating that clean production policy integration can reconcile environmental and economic objectives. These findings provide empirical evidence supporting circular economy strategies for air pollution mitigation in industrializing regions.

## 1. Introduction

China’s rapid industrialization and urbanization over recent decades have spurred unprecedented economic growth, but have led to significant air pollution challenges, with profound implications for public health and sustainable development. Addressing these issues is critical for achieving long-term environmental and economic goals [1,2,3,4]. In this context, fine particulate matter (PM_2.5_) has emerged as the primary pollutant affecting air quality, with its pollution trends and harmful effects intensifying significantly over the past decade, attracting widespread attention from academia and society [3,5]. Severe PM_2.5_ pollution is highly detrimental to human health, being linked to numerous adverse impacts. For instance, it affects the respiratory, cardiovascular, and immune systems, reduces visibility (increasing traffic accidents), impacts climate change, and damages ecosystems [6]. 4.2 million deaths annually are attributed to PM_2.5_ exposure [7]. Sidell et al. (2022) found that PM_2.5_ exposure is associated with an increased incidence of COVID-19, with PM_2.5_ pollution influencing disease transmission during the pandemic [8]. Parental exposure to real-world PM_2.5_ has also been observed to cause intergenerational primary hypogonadism in a gender-selective manner [9]. An analysis of PM_2.5_ pollution’s impact on the health of adults aged 45 and above in Chinese prefecture-level cities revealed that PM_2.5_ significantly reduces self-rated health, increases the risk of chronic diseases, and elevates the probability of mental depression [10]. Despite substantial progress in air quality management, China continues to face significant PM_2.5_ pollution challenges, with particularly elevated concentrations persisting during autumn and winter seasons [11,12]. Therefore, advancing air pollution control in Shandong province is not only critical for improving air quality within the province but also essential for reducing cross-regional pollutant transmission and supporting air quality improvement in surrounding areas such as Beijing [13]. Systematically analyzing the spatiotemporal patterns of PM_2.5_ and its influencing factors holds significant theoretical and practical value for formulating scientific, targeted pollution prevention strategies and continuously improving regional air quality [14].

Existing research on PM_2.5_ pollution has explored its spatiotemporal distribution characteristics [15,16]. Yu et al. (2021) examined the spatiotemporal variations and driving factors of PM_2.5_ concentrations in Shandong province from 2014 to 2017 [17]. Xu et al. (2022) studied PM_2.5_ spatial distribution in several Chinese regions, noting that most areas maintain relatively high PM_2.5_ concentrations with distinct spatial characteristics [18]. Research on PM_2.5_-influencing factors has also gained attention [19,20,21]. Li et al. (2019) highlighted that meteorological factors, including precipitation, wind speed, wind direction, and temperature, are closely related to the formation, intensification, diffusion, and dilution of air pollution [22]. Guo et al. (2024) identified precipitation, average air pressure, and average air temperature as key meteorological factors affecting air quality variations [23]. Yang et al. (2017) revealed significant regional differences in the relationship between PM_2.5_ concentrations and meteorological factors across China [24]. In northern China and Urumqi, relative humidity correlates positively with PM_2.5_ concentrations, whereas this relationship is negative in other regions; regarding wind speed, Hainan Island is the only region without a significant negative correlation with PM_2.5_, while such an inhibitory relationship exists elsewhere [24]. Deng et al. (2022) found that precipitation and temperature exert significant inhibitory effects on PM_2.5_ pollution in the BTH urban agglomeration, whereas meteorological parameters such as wind speed, relative humidity, and sunshine duration exhibit synergistic intensifying effects [25].

Current research on PM_2.5_ pollution predominantly examines short-term spatiotemporal distribution patterns in individual cities or regions, emphasizing meteorological influences while neglecting comprehensive analyses of socioeconomic drivers. To address this gap, this study investigates the long-term, large-scale spatiotemporal dynamics of PM_2.5_ pollution in Shandong province, China, systematically evaluating its distribution characteristics and variability across annual, seasonal, and monthly scales. Furthermore, we quantify the dual impacts of socioeconomic and meteorological factors, providing a holistic perspective on the underlying drivers of PM_2.5_ pollution. Our findings offer critical insights for sustainable air quality management and policy formulation, aligning with the goals of resource conservation and environmental sustainability.

## 2. Data and Methods

### 2.1. Study Area

**Figure 1:**
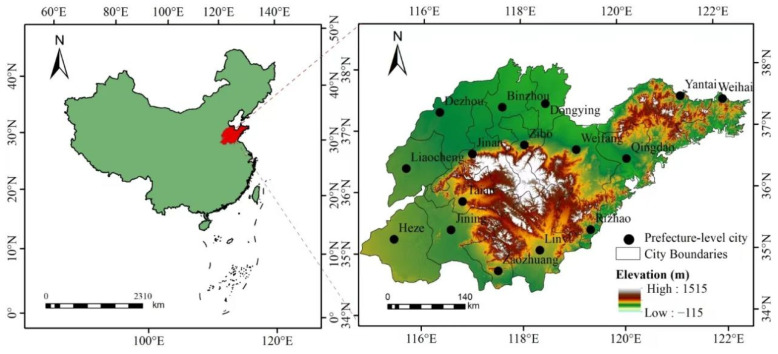
Location map of Shandong province.

Shandong province is a critical economic and ecological hub in eastern China, situated along the Yellow River downstream (Figure 1). From 2014 to 2023, Shandong experienced rapid socioeconomic growth, with its GDP increasing from 5.94 trillion yuan to 9.2 trillion yuan, a permanent population reaching 101.23 million, and electricity consumption surging to 796.56 billion kWh. These trends underscore the province’s escalating energy demands and associated environmental pressures, making it an ideal case study for analyzing the interplay between economic activity and air pollution.

### 2.2. Data Collection and Processing

This study utilized PM_2.5_ concentration data (2014–2023) obtained from the Ministry of Ecology and Environment of China (MEE) (https://www.mee.gov.cn/), encompassing 16 ground-based monitoring stations distributed across all 16 prefectural-level cities in Shandong Province. Meteorological data and socioeconomic indicators were acquired from the Shandong Provincial Meteorological Bureau (CMA) (http://sd.cma.gov.cn/), encompassing 16 meteorological stations and Shandong Provincial Bureau of Statistics (http://tjj.shandong.gov.cn/), respectively. Daily monitoring data were aggregated to derive monthly, seasonal, and annual average PM_2.5_ concentrations. This temporal resolution enables comprehensive analysis of long-term trends in particulate pollution. The maps are generated using ArcGIS Pro 2.5.

### 2.3. Analytical Methods

#### 2.3.1. Spatial Interpolation and Cluster Analysis

We employed ordinary Kriging interpolation to analyze the spatiotemporal distribution patterns of PM_2.5_ concentrations across annual, seasonal, and monthly timescales (Table 1) [26]. This geostatistical approach provides optimal unbiased estimates for unsampled locations while quantifying spatial autocorrelation. For meteorological and socioeconomic data analysis, we implemented K-means clustering to identify homogeneous groups within the multivariate dataset. The cluster centroids were calculated as:

$$\mu_{kj}=\frac{1}{N_{k}}\overset{N_{k}}{\underset{i=1}{\sum }}x_{kij}$$

where *μkj* represents the centroid of cluster *k* for variable *j*, *N_k_* denotes the number of observations in cluster *k*, and *x_kij_* is the value of variable *j* for observation *i* in cluster *k*.

#### 2.3.2. Statistical Analysis

All variables were standardized (z-score normalization) prior to correlation analysis to ensure comparability across different measurement scales. We quantified variable relationships using Pearson’s correlation coefficient:

$$r=\frac{\sum_{i=1}^{n}(x_{i}-\overset{–}{x})\left(y_{i}-\overset{–}{y}\right)}{\sqrt{\sum_{i=1}^{n}{\left(x_{i}-\overset{–}{x}\right)}^{2}\sum_{i=1}^{n}{\left(y_{i}-\overset{–}{y}\right)}^{2}}}$$

where *r* represents the correlation coefficient between variables *x* and *y*, with values ranging from −1 to 1.

#### 2.3.3. Geodetector Analysis

We applied Geodetector method to examine spatial heterogeneity and identify driving factors of PM_2.5_ pollution (Table 1) [27,28]. The factor detection function quantifies explanatory power as:

$$q=1-\frac{\sum_{h=1}^{L}N_{h}\sigma_{h}^{2}}{N\sigma^{2}}$$

where *q* ∈ [0, 1] represents the explanatory power of factor *X* on PM_2.5_. *N* and *Nh* denote sample sizes for the entire study area and stratum *h* (partitioned by factor *X*), respectively. *σ*^2^ and *σh*^2^ represent the variances of *X* in the entire area and stratum *h.* Higher *q* values indicate greater influence of the explanatory factor on PM_2.5_ spatial distribution.

## 3. Results

### 3.1. Annual Spatiotemporal Variation Characteristics of PM_2.5_ Concentrations

**Figure 2:**
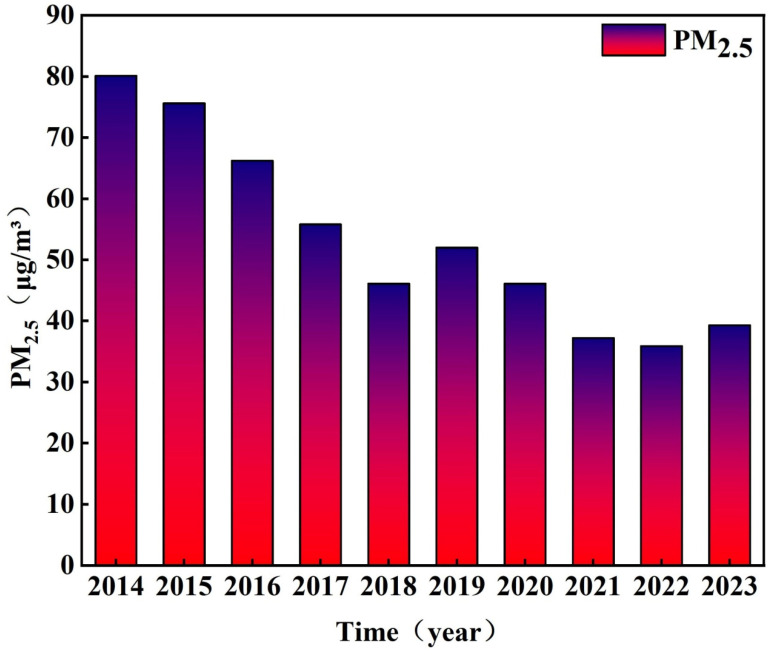
Annual variation trend of PM_2.5_ concentrations in Shandong province from 2014 to 2023.

From 2014 to 2023, Shandong province experienced a statistically significant year-on-year decline in PM_2.5_ concentrations, demonstrating substantial improvements in air quality. China achieved remarkable progress in PM_2.5_ reduction over the past decade. During the COVID-19 containment period in 2020, stringent mobility restrictions led to reduced economic activity and significantly lower emissions, resulting in anomalously low PM_2.5_ levels across all chemical components [29]. In the post-pandemic economic recovery phase, the rebound in industrial production, energy demand, and transportation activities initially increased fossil fuel consumption and emissions. However, through optimized energy structures, expanded clean energy adoption, and strengthened pollution control measures, PM_2.5_ pollution levels subsequently declined, following a “decline-rebound-decline” trajectory. Despite these fluctuations, Shandong province maintained an overall downward trend in annual average PM_2.5_ concentrations (Figure 2). Specifically, the annual mean PM_2.5_ concentration decreased from 80.1 μg/m^3^ in 2014 to 39.3 μg/m^3^ in 2023, representing a cumulative reduction of 40.8 μg/m^3^ and a decrease of over 50%.

To accurately reveal the spatiotemporal distribution patterns of PM_2.5_ pollution in Shandong Province, this study employed Kriging interpolation (with an exponential variogram model) as the primary spatial interpolation technique, while the Inverse Distance Weighting (IDW) method was introduced for comparative validation to assess the applicability and accuracy of different interpolation approaches. Based on cross-validation analysis, the root mean square error (RMSE) of the Kriging method was significantly lower than that of IDW, indicating its superior capability in quantifying spatial distribution differences of PM_2.5_ concentrations with reduced estimation errors and improved data fitting precision (Table 2). Furthermore, the regression slope of the Kriging interpolation was closer to unity, suggesting higher consistency between the interpolated results and the actual monitoring data. This demonstrates that Kriging more reliably reflects the distribution characteristics and variation trends of PM_2.5_ concentrations across both temporal and spatial dimensions. Considering the comprehensive accuracy verification metrics and the reliability of the results, the Kriging interpolation method (with an exponential model) is more suitable for quantitatively analyzing the spatiotemporal distribution features of PM_2.5_ pollution in Shandong Province in this study, thereby providing a scientific methodological foundation for subsequent investigation into the evolution of pollution patterns.

**Figure 3:**
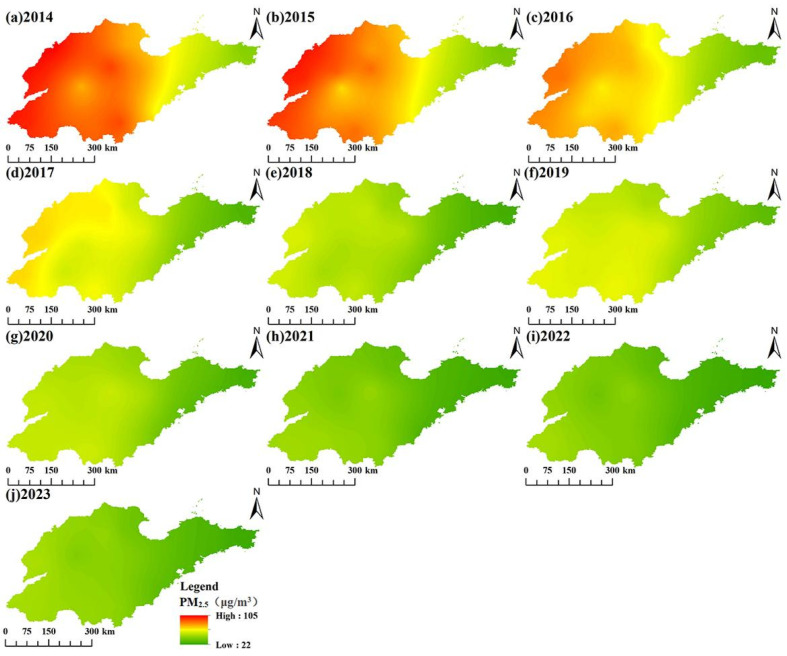
Annual spatial distribution of PM_2.5_ concentrations in Shandong province from 2014 to 2023.

PM_2.5_ concentrations in Shandong province exhibited pronounced spatial heterogeneity, with distinct west–east variation (Figure 3). Our analysis revealed a dominant distribution pattern: a marked west–east gradient, with higher concentrations in western regions and lower levels in eastern coastal areas. The highest PM_2.5_ concentrations consistently occurred in southwestern and central urban clusters, particularly encompassing Dezhou, Liaocheng and Heze. In contrast, the eastern coastal cities of Qingdao, Yantai, and Weihai maintained comparatively cleaner air. This spatial pattern reflected a progressive worsening of air quality from the eastern peninsula toward inland western regions, with pollution severity following the order: western > central > eastern Shandong. The significant improvement in air quality after 2018 is related to the implementation of the Clean Air Action Policy.

### 3.2. Seasonal Spatiotemporal Variation Characteristics of PM_2.5_ Concentrations

**Figure 4:**
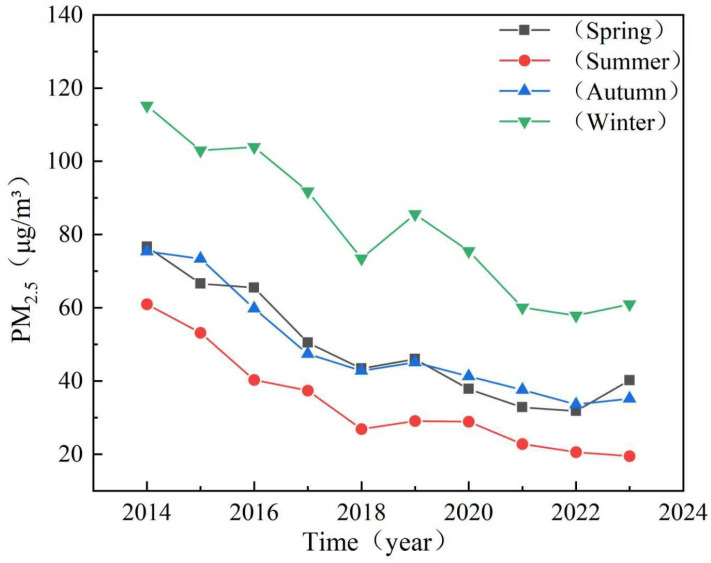
Seasonal variation of PM_2.5_ concentrations in Shandong province from 2014 to 2023.

**Figure 5:**
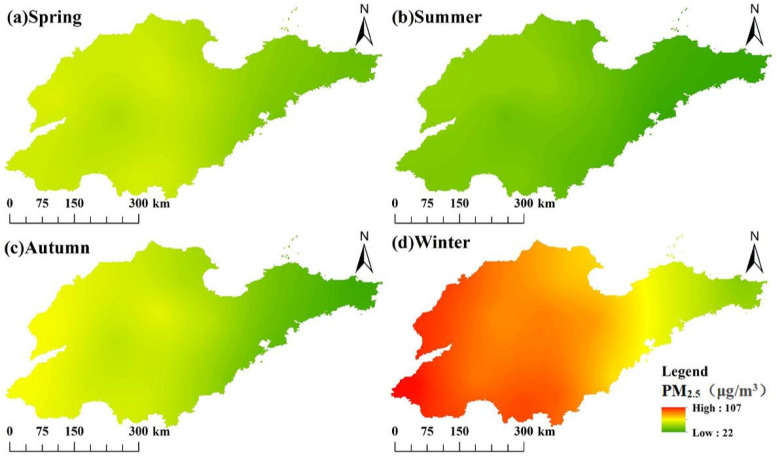
Seasonal spatial distribution of PM_2.5_ concentrations.

This study identified clear seasonal and spatial patterns in PM_2.5_ concentrations across Shandong province, with winter concentration (82.7 μg/m^3^) and summer concentration (34.0 μg/m^3^) driven by seasonal variations in atmospheric stability, heating emissions, and dispersion conditions (Figure 4). Temporal analysis indicated a consistent distribution feature of “higher in winter and lower in summer”. The winter peak is related to increased heating emissions and stable meteorological conditions (such as lower boundary layer height and higher inversion frequency), while the summer minimum is related to enhanced atmospheric diffusion and precipitation clearance. The high concentration areas in winter expand and intensify towards the west, while the more uniform and lower concentrations observed throughout the province in summer. The results demonstrated a consistent concentration gradient between inland and coastal regions, with highest pollution levels in southwestern and central urban areas (Dezhou-Liaocheng-Heze cluster) due to coal-dependent heating systems and topographic effects, while eastern coastal cities (Qingdao-Yantai-Weihai) benefit from marine atmospheric influences (Figure 5). The observed patterns highlighted the critical influence of both meteorological factors and energy infrastructure on air quality dynamics, suggesting that targeted regional policies addressing winter heating practices and spatial emission management could significantly reduce population exposure to extreme particulate pollution.

### 3.3. Monthly Spatiotemporal Variation Characteristics of PM_2.5_ Concentrations

**Figure 6:**
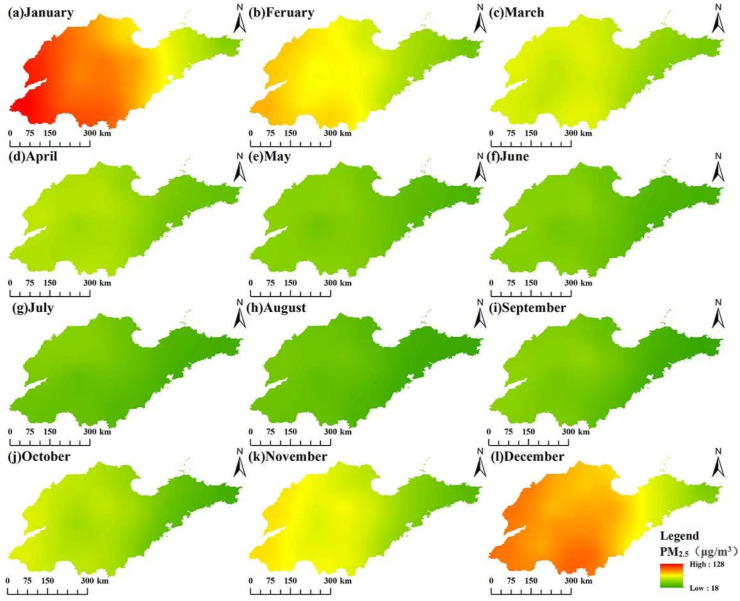
Monthly spatial distribution of PM_2.5_ concentrations.

**Figure 7:**
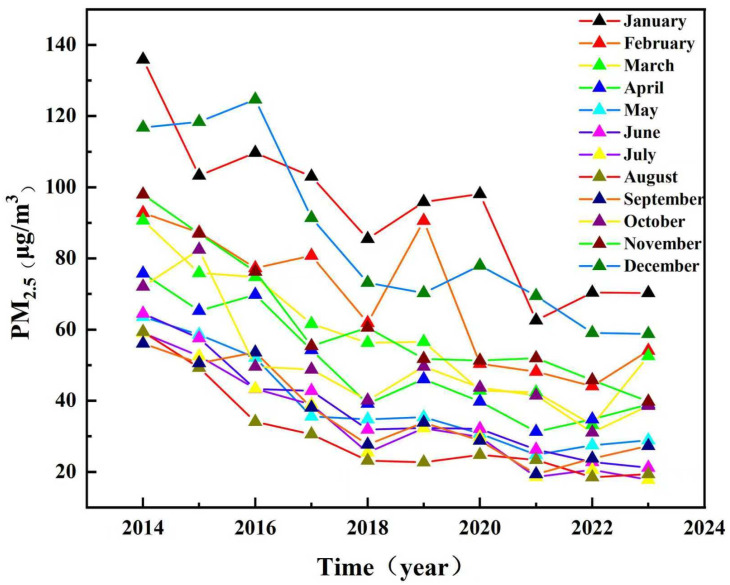
Monthly variation of PM_2.5_ concentrations in Shandong province from 2014 to 2023.

Monthly PM_2.5_ concentrations in Shandong province (2014–2023) exhibited consistent spatial (west–east gradient) and temporal (winter-summer contrast) patterns (Figure 6). PM_2.5_ concentrations peaked in January and reached a trough in August. A steady declining trend was observed from January to August (Figure 7). Due to the increase in aerosol hygroscopicity during winter, PM_2.5_ concentrations rose in November and December. Notably, January concentrations decreased from 2014 to 2023, demonstrating the cumulative impact of China’s air quality policies. The persistent west–east disparity underscored the interplay between regional emission sources (e.g., western coal heating) and geographic advantages (eastern marine dispersion). These trends highlighted both the effectiveness of long-term emission controls and the need for seasonally targeted measures, particularly for winter heating and industrial sources.

### 3.4. Variation Characteristics in the Centers of Gravity of PM_2.5_ Concentrations

**Figure 8:**
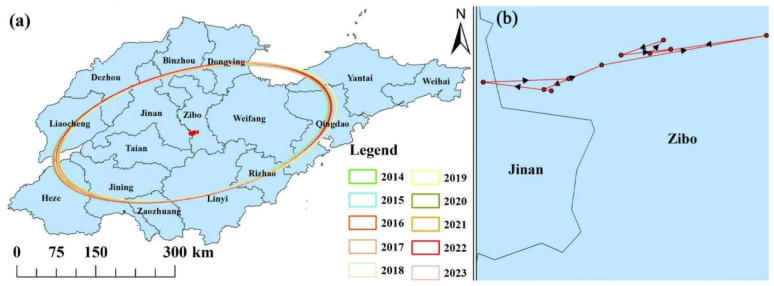
The standard deviation ellipses and trajectory of PM_2.5_ pollution centers of gravity in Shandong province from 2014 to 2023. (**a**) Standard deviation ellipses and gravity centers of PM_2.5_ concentrations over 2014–2023, (**b**) PM_2.5_ gravity center migration trajectory over 2014–2023. Circles denote the PM_2.5_ pollution center of gravity, triangles indicate the direction of PM_2.5_ pollution trajectory change, and lines represent the path of the center of gravity.

The standard deviation ellipse approach was used to quantify the local spatial characteristics of PM_2.5_ concentrations. The major axis of the ellipse expressed the distribution direction of PM_2.5_ concentrations, and the short half-axis represented the distribution range of PM_2.5_ concentrations. The main distribution of PM_2.5_ concentrations in Shandong was aligned in the northeast-southwest direction (Figure 8a). Our gravity model analysis revealed that PM_2.5_ pollutions in Shandong province exhibited distinct spatial clustering and temporal migration patterns from 2014 to 2023. The pollution centroid displayed a characteristic phased migration, initially shifting westward before transitioning to accelerate northeastward movement. The center of gravity of PM_2.5_ in Shandong province migrated to the southwest overall during 2014–2023. However, the center of gravity was consistently located in a stable region southwest of Zibo, demonstrating remarkable spatial stability despite these migration trends (Figure 8b).

### 3.5. Interaction Effects of Meteorological Factors on PM_2.5_ Concentrations

**Figure 9:**
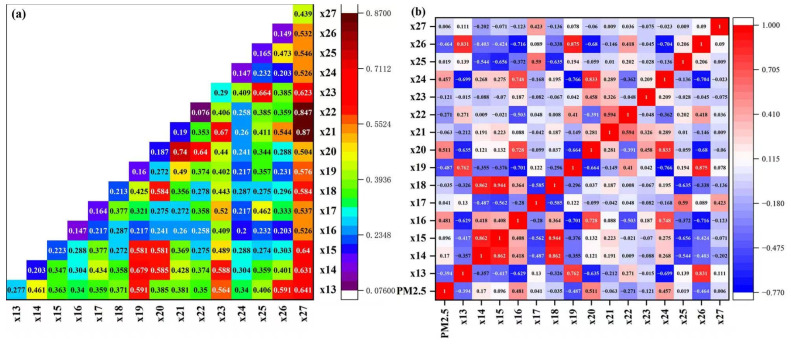
Interaction detection values and correlation coefficients between PM_2.5_ and meteorological factors. (**a**) Interaction detection of PM_2.5_ and meteorological factors (**b**) Correlation coefficients of PM_2.5_ and meteorological factors (‘x13—Maximum wind speed’, ‘x14—Minimum air pressure’, ‘x15—Maximum air pressure’, ‘x16—Maximum air temperature’, ‘x17—Precipitation’, ‘x18—Average air pressure’, ‘x19—Average wind speed’, ‘x20—Average air temperature’, ‘x21—Average water vapor pressure’, ‘x22—Average relative humidity’, ‘x23—Average minimum air temperature’, ‘x24—Average maximum air temperature’, ‘x25—Number of days with daily precipitation ≥ 0.1 mm’, ‘x26—Maximum wind speed’, ‘x27—Maximum daily precipitation’). All interactions significant at *p* < 0.001 level.

The geodetector (interaction detector) analysis revealed significant synergistic effects among meteorological drivers of PM_2.5_ pollution through interaction detection (Figure 9a). Three key interaction patterns were identified: (1) thermodynamic-humidity interactions (air temperature × water vapor pressure, q = 0.740; air temperature × relative humidity, q = 0.640; average minimum air temperature ×maximum daily precipitation, q = 0.623) demonstrating how warm, humid conditions promote secondary aerosol formation while enhancing atmospheric stability, accounting for 64–74% of PM_2.5_ variability; (2) dynamic dispersion mechanisms (wind speed × minimum pressure, q = 0.679; wind speed × maximum daily precipitation, q = 0.576, wind speed ×average minimum air temperature, q = 0.564; wind speed ×relative humidity, q = 0.374) revealing frontal systems’ role in explaining 58–68% of seasonal transition period PM_2.5_ changes; and (3) precipitation-humidity synergy (maximum daily precipitation × water vapor pressure, q = 0.870; maximum daily precipitation × relative humidity, q = 0.847) highlighting humid conditions’ dual function in facilitating aerosol growth while enhancing wet deposition efficiency during precipitation events. In short, the interactions between air temperature, wind speed, precipitation, and relative humidity significantly amplified their respective impacts.

This study also revealed that maximum air temperature (r = 0.481) and average air temperature (r = 0.511) exhibited moderate positive correlations with PM_2.5_ concentrations (Figure 9b). These relationships may be driven by two mechanisms: (1) enhanced photochemical reactions under elevated temperatures, promoting secondary aerosol formation, and (2) increased atmospheric stability under high-temperature conditions, which suppresses vertical dispersion and leads to PM_2.5_ accumulation in the atmosphere. These findings underscored the need for targeted PM_2.5_ control measures during high-temperature episode. In contrast, significant negative correlations were observed between PM_2.5_ concentrations and both maximum wind speed (r = −0.394) and average wind speed (r = −0.487). This demonstrated the critical role of wind in pollutant dispersion, as higher wind speeds enhance horizontal advection and dilution of PM_2.5_. Notably, periods of elevated wind speeds often coincided with synoptic-scale weather changes (e.g., cold front passages), which may further reduce PM_2.5_ through wet deposition via associated precipitation events. These results highlight wind speed as a key meteorological determinant of PM_2.5_ variability. Air pressure parameters showed minimal influence, with weak correlations for both minimum (r = 0.170) and maximum air pressure (r = 0.096). This suggested that air pressure variations alone had negligible direct effects on PM_2.5_ concentrations, likely because pressure changes were typically coupled with other meteorological factors (e.g., wind, precipitation) that dominated pollutant dispersion and removal processes. The overall correlation between average relative humidity and PM_2.5_ concentration was also negative (r= −0.271). There was a positive correlation between precipitation and PM_2.5_ (r= 0.041). Collectively, these results demonstrated that air temperature and wind speed were the dominant meteorological drivers of PM_2.5_ variability, while air pressure played a relatively minor role in directly influencing particulate pollution levels.

### 3.6. Interaction Effects of Socioeconomic Factors on PM_2.5_ Concentrations

**Figure 10:**
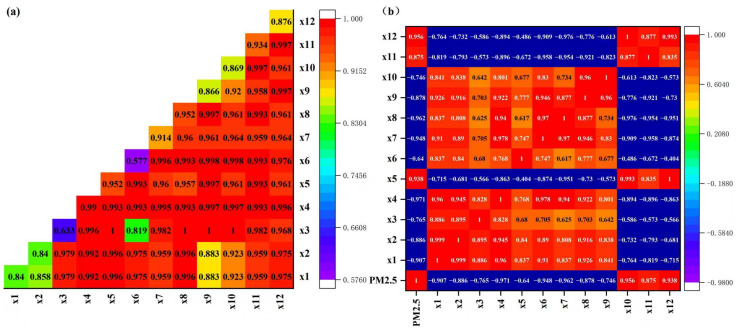
Interaction detection values and correlation coefficients (r) between PM_2.5_ and socio-economic factors. (**a**) Interaction effects between socioeconomic factors on PM_2.5_ concentrations; (**b**) Correlation coefficients of PM_2.5_ and socio-economic factors (‘x1—GDP’, ‘x2—Per capita GDP’, ‘x3—Secondary industry production’, ‘x4—Tertiary industry production’, ‘x5—Sulfur dioxide emissions’, ‘x6—Agriculture’, ‘x7—Forestry’, ‘x8—Population’, ‘x9—Electricity consumption’, ‘x10—Industrial electricity consumption’, ‘x11—Nitrogen oxides emissions’, ‘x12—Particulate matter emissions’). All interactions significant at *p* < 0.001 level.

The interaction detector analysis revealed complex interdependencies among socioeconomic drivers of PM_2.5_ pollution in Shandong province (Figure 10a). The q values represented explanatory power of two-factor combinations. The interaction effects between per capita GDP and tertiary industry production showed exceptionally strong explanatory power (q = 0.992), with PM_2.5_ concentrations exhibiting an inverted U-shaped relationship consistent with the Environmental Kuznets Curve (EKC). This suggested that economic growth beyond a threshold (approximately $6000 per capita) corresponded to improved air quality, likely due to structural shifts toward tertiary industries (sectoral transition effects). The Geodetector analysis revealed particulate matter (PM) emissions as the most influential factor in PM_2.5_ pollution dynamics, exhibiting exceptionally strong explanatory power and interaction between PM and co-emitted pollutants. PM-SO_2_ synergy (q = 0.961) suggested shared combustion sources. PM-NO_x_ interaction (q = 0.997) indicated transportation sector contributions. The combined effect of SO_2_ and NO_x_ emissions accounted for 99.3% of PM_2.5_ variability (q = 0.993), indicating their dominant role as precursor pollutants. Interestingly, the q values (tertiary industry production × SO_2_, q = 0.993; tertiary industry production × PM, q = 0.995) of the effects of the on PM_2.5_ were relatively high, revealing the critical role of industrial structure in air pollution dynamics. The coupled effect of multiple socioeconomic factors on PM_2.5_ was greater than that of a single factor. These findings provided empirical evidence that coordinated economic-environmental policies could effectively decouple development from air pollution, for example, industrial emission synergies, energy efficiency improvements, and structural economic transformation.

The evaluated socio-economic factors exhibited significant impacts on PM_2.5_ concentrations (Figure 10b). Notably, the correlation coefficient (r) between tertiary industry production and PM_2.5_ was −0.971, suggesting that economic restructuring had contributed to improved air quality. The r between per capita GDP and PM_2.5_ was −0.886. In contrast, particulate matter emissions (r = 0.956) and sulfur dioxide emissions (r = 0.938) showed strong positive correlations with PM_2.5_, indicating that reductions in these emissions had effectively lowered PM_2.5_ levels. A strong negative correlation (r = −0.746) was observed between industrial electricity consumption and PM_2.5_, likely reflecting industrial efforts to enhance energy efficiency and adopt cleaner technologies [30]. Furthermore, the high coefficient of determination (R^2^) value (0.805) between per capita GDP and PM_2.5_ concentrations aligned with the EKC hypothesis [31], supporting the notion that environmental degradation initially intensified with economic growth but eventually improved after reaching a certain income threshold. These findings highlighted that pollutant emissions and industrial structure adjustments were key determinants of PM_2.5_ concentrations. To mitigate PM_2.5_ pollution effectively, policymakers should prioritize stringent emission controls, promote industrial technological advancements, and incentivize sustainable economic transitions.

## 4. Discussion

### 4.1. Policy Impact

China implemented three phases of clean air actions in 2013, 2018, and 2021, including Air Pollution Prevention and Control Action Plan, Three-Year Action Plan for Blue Sky Defense War, and Deepening Pollution Prevention Campaign. China’s air quality governance has evolved through three strategic phases of clean air actions, driving a 28% reduction in national PM_2.5_ concentrations (2014–2018) [32]. Shandong has shifted from high-speed growth to high-quality development, entering a pivotal stage of transforming its development approach, upgrading its economic structure, and fostering new growth drivers. Shandong province’s implementation of these policies through its clean air actions and Blue Sky Defense War Action Plans demonstrates the effectiveness of integrated emission control measures. Our analysis reveals significant improvements in air quality, with the annual mean PM_2.5_ concentration decreasing from 80.1 μg/m^3^ in 2014 to 39.3 μg/m^3^ in 2023. This represents an absolute reduction of 40.8 μg/m^3^ (50.9% decrease), but it do not achieve WHO Interim Target 3 (35 μg/m^3^). We recommend implementing integrated SO_2_-NO_x_ abatement strategies to maximize synergistic emission reductions (demonstrated q = 0.993), while accelerating low-carbon transition of industrial power generation through renewable energy penetration and circular economy measures to achieve absolute decoupling of energy use from PM_2.5_ emissions. Greenhouse gases and atmospheric pollutants share the same root and source, and achieving carbon neutrality in Shandong province will help achieve the goal of improving air quality.

The components of PM_2.5_ in Shandong Province consist of primary pollutants, secondary inorganic aerosols (SIA) (the dominant components of PM_2.5_), and secondary organic aerosols (SOA). Primary pollutants mainly include dust, black carbon (BC), inorganic ions, organic carbon (OC), and metal particles, which are directly emitted from sources such as industrial processes, vehicle exhaust, biomass burning, and road dust. As a major industrial and agricultural province in China, Shandong’s high-intensity coal consumption and agricultural activities provide ample precursors (SO_2_, NO_x_, NH_3_) for the formation of SIA. Secondary inorganic ions (i.e., SO_4_^2−^, NO_3_^−^, NH_4_^+^) are the largest component of PM_2.5_. Sulfates are primarily derived from the oxidation of sulfur dioxide (SO_2_) emitted from coal combustion, particularly from industrial coal use and power plants. Nitrates mainly originate from the transformation of nitrogen oxides (NO_x_) released by motor vehicles, coal combustion, and industrial processes. Ammonium salts are largely sourced from agricultural ammonia emissions (e.g., fertilizer application, livestock farming). Ammonia acts as a neutralizing agent, combining with sulfuric and nitric acids to form stable ammonium sulfate and ammonium nitrate particles.

SOA is formed through the photochemical reactions of volatile organic compounds (VOCs) in the atmosphere. The precursor VOCs originate from various sources, including fossil fuel combustion, biomass burning, industrial solvent use, petrochemical and chemical processes, and motor vehicle emissions. It is crucial to note that SOA formation requires not only VOCs but also oxidants. Therefore, the synergistic control of NO_x_ and VOCs is essential to effectively suppress the concurrent formation of SOA.

### 4.2. Relationship Between Meteorological Factors and PM_2.5_

Meteorological factors (air temperature and precipitation) have an impact on improving AQI in most regions of China. Air temperatures between approximately 8–17 °C exacerbate pollution, while temperatures above or below this range improve air quality [33]. The removal and dilution of particulate matter may be related to rainfall and wind speed. When the wind is strong, pollutants are quickly dispersed, leading to a reduction in local PM_2.5_ concentrations [34]. This indicates that the physical mechanism of atmospheric advective dispersion plays a universally dominant role in determining particulate matter concentrations, both in Vietnam and in Shandong Province, China. Similarly, air pressure is a key factor affecting PM_2.5_ pollution [35]. Changes in air pressure can cause air movement, altering the direction and speed of air flow, which in turn leads to the convergence or dispersion of PM_2.5_ across different regions, thereby affecting the levels of PM_2.5_ concentrations in various areas. Moreover, anomalies in the pressure field can also exacerbate regional pollution by inhibiting vertical diffusion, resulting in increased PM_2.5_ concentrations, especially during the winter heating period [36,37]. These studies indicate that anomalies in air pressure can exacerbate pollution by suppressing vertical dispersion. However, our results suggest that in Shandong Province, the influence of air pressure changes may be largely masked or coupled with other more potent concurrent factors (such as wind and precipitation), resulting in a relatively less significant independent contribution. This conclusion is highly consistent with the analysis results of meteorological impact factors on PM_2.5_ in Shandong province in this study. Nguyen et al., (2024) found that among numerous meteorological factors, temperature, wind speed, and air pressure have the most significant impacts on PM_2.5_ concentrations in Vietnam, collectively influencing the diffusion, transmission, and accumulation processes of pollutants [38]. It was found that temperature and radiation have the greatest impact on PM_2.5_ concentrations, followed by humidity and wind speed, then air pressure and wind direction. PM_2.5_ pollution events are more common under conditions of high humidity, high air pressure, low temperature, low radiation, and low wind speed. Higher temperatures can inhibit PM_2.5_ concentrations because they facilitate the volatilization and diffusion of pollutants, while lower temperatures can exacerbate pollution due to the enhancement of secondary reactions [39]. As a province characterized by high industrial emissions and intensive agricultural activities, Shandong’s atmosphere is rich in precursors such as SO_2_, NO_x_, and VOCs. Under these conditions, high temperatures and intense sunlight significantly accelerate photochemical reactions, promoting the formation of secondary inorganic/organic aerosols. This effect, combined with the suppression of dispersion, outweighs the potential dilution effect of high temperatures, ultimately leading to a net positive correlation. These findings align with our comprehensive analysis of meteorological drivers of PM_2.5_ pollution in Shandong province.

The generation and dissipation of air pollutants undergo highly complex atmospheric chemical/physical processes, and meteorological conditions ultimately determine the concentration of PM_2.5_ near the ground by influencing the processes of pollutant emissions (secondary formation), dispersion, transport, and removal.

High temperatures and intense sunlight accelerate photochemical reactions in the atmosphere. This causes precursor pollutants (such as SO_2_, NO_x_, and VOCs) to react more rapidly, generating substantial amounts of SIA, including sulfates, nitrates, and ammonium salts, as well as SOA. High temperatures, especially on calm, sunny summer days, often lead to a stable atmospheric stratification. Temperature inversions suppress vertical air convection, preventing pollutants generated near the surface from dispersing upward, which leads to pollutant accumulation.

Winds blow airborne pollutants away. Higher wind speeds significantly enhance the horizontal transport (advection) and dilution of airborne pollutants, thereby reducing local PM_2.5_ concentrations. Precipitation removes particles from the air through wet deposition (scavenging).

Higher humidity can cause more steam to adhere to particulate matter (PM) and significantly increase the size and mass concentration of particulate matter (PM), resulting in increased hygroscopicity and accumulation of PM_2.5_. Under certain conditions, high humidity can also promote the formation of secondary aerosols (for example, the aqueous-phase oxidation of SO_2_ in droplets to form sulfate), potentially increasing PM_2.5_ concentrations. Our results show an overall negative correlation between humidity and PM_2.5_, indicating that, during the study period, the overall effect of removal processes associated with high humidity (such as precipitation) was stronger than its effect on promoting aerosol formation.

High temperature and high humidity conditions collectively promote the formation of secondary inorganic aerosols such as sulfate (SO_4_^2−^), nitrate (NO_3_^−^), and ammonium (NH_4_^+^). Elevated temperatures accelerate the gaseous oxidation rate of sulfur dioxide (SO_2_). Simultaneously, high relative humidity provides favorable conditions for the aqueous-phase oxidation of SO_2_ (e.g., by oxidants such as O_3_ and H_2_O_2_), which is a critical pathway for sulfate formation in Shandong Province. Temperature influences the gas-particle partitioning of ammonium nitrate (NH_4_NO_3_). Although high temperatures theoretically promote the volatilization of NH_4_NO_3_, under the specific ammonia (NH_3_)-rich conditions in Shandong, combined with high humidity, the heterogeneous conversion process of nitrogen oxides (NO_x_) to nitrate is enhanced. This may maintain nitrate concentrations at relatively high levels, particularly during transitional seasons. Higher temperatures also accelerate the volatilization of volatile organic compounds (VOCs) and their photochemical reactions in the atmosphere, generating semi-volatile and low-volatility products. These subsequently form secondary organic aerosols (SOA) through gas-particle partitioning, aqueous-phase reactions, or hygroscopic growth under high humidity conditions.

When wind speed is low and under the influence of high-pressure systems, the atmospheric boundary layer becomes stable, and vertical diffusion capacity is significantly weakened. Pollutants are compressed into a shallow layer near the surface, unable to effectively disperse and dilute, leading to the rapid accumulation of PM_2.5_.

Our results indicate a weak positive correlation between precipitation and PM_2.5_. A possible explanation for this is seasonal confounding factors. The period with the highest PM_2.5_ concentrations throughout the year is winter (due to heating emissions), which is also a season with typically low precipitation. In contrast, summer experiences high precipitation and other favorable meteorological conditions (such as stronger winds and enhanced atmospheric dispersion capacity), resulting in lower PM_2.5_ concentrations. Therefore, annual statistical analysis may show a weak positive correlation between precipitation and PM_2.5_. However, this does not mean that precipitation exacerbates pollution; rather, it reflects the influence of different dominant factors across seasons.

Therefore, meteorological conditions exert short-term influences on PM_2.5_. Wind speed and direction directly affect the horizontal dispersion capacity of pollutants. Calm or stagnant weather is the primary cause of heavy pollution episodes. The presence of temperature inversion layers strongly suppresses vertical dispersion, leading to the rapid accumulation of pollutants near the ground. High humidity promotes heterogeneous chemical reactions that form secondary particles, while precipitation effectively removes particles through wet deposition. During daytime, solar heating elevates the planetary boundary layer, providing a larger volume for pollutant dilution; at night, a lower boundary layer leads to increased pollutant concentrations. These factors are the primary short-term drivers responsible for diurnal variations in PM_2.5_ concentrations and the occurrence of pollution episodes.

### 4.3. Relationship Between Socio-Economic Factors and PM_2.5_

Comprehensive analyses of socioeconomic impacts on PM_2.5_ pollution demonstrate that emission control measures (2013–2020) played a dominant role in air quality improvement compared to meteorological factors, particularly in the BTH region where PM_2.5_ concentrations decreased 57% from 106 to 45.1 μg/m^3^, with emission reductions accounting for 95% (58 μg/m^3^) of this decline [40]. The observed Environmental Kuznets Curve relationship reveals complex economic linkages, where GDP growth initially exacerbates but ultimately reduces PM_2.5_ pollution through structural economic transitions and policy interventions [41]. Our findings demonstrate that regional development strategies significantly influence PM_2.5_ mitigation trajectories. (1) We validate the Environmental Kuznets Curve (EKC) relationship in Shandong (r^2^ = 0.805), where economic growth initially increases but ultimately reduces PM_2.5_ concentrations after reaching a turning point [42,43]. (2) The development of the tertiary industry is conducive to improving air pollution PM_2.5_. (3) synergistic pollution control where each 1% increase in renewable energy penetration enhances SO_2_ abatement effectiveness by 0.8–1.5 percentage points [44], with the strong SO_2_-PM_2.5_ correlation (r = 0.938) confirming the co-benefits of combining clean energy transition with end-of-pipe controls. These results provide policymakers with quantitative evidence that coordinated energy transition and industrial upgrading strategies can simultaneously improve air quality and maintain economic growth.

Pollution control policies, industrial structure, and socioeconomic development have long-term impacts on PM_2.5_. Stringent clean air policies drive the sustained decline of PM_2.5_ concentrations through various pathways, such as promoting the transition to cleaner energy and transportation, and deepening industrial governance. An industrial structure characterized by high energy consumption and high pollution directly determines the baseline emission levels of precursors like sulfur dioxide (SO_2_) and nitrogen oxides (NO_x_). The pace of economic growth drives the overall emissions of primary particulate matter, nitrogen oxides, and volatile organic compounds (VOCs).

Therefore, from a long-term perspective, it is essential to optimize the industrial and energy structures and promote a green transition to reduce pollutant emissions at the source. In the short term, it is necessary to enhance air quality forecasting capabilities and precisely activate emergency control measures when unfavorable meteorological conditions are predicted. This approach helps to “shave the peak and slow the buildup,” thereby preventing the occurrence of heavy pollution events.

Although data limitations prevented the inclusion of key socioeconomic factors such as occupation patterns, technological advancement, population density, green space distribution and income levels in this study, future research should incorporate these variables to better understand their contextual impacts on urban air pollution across different global regions.

## 5. Conclusions and Future Work

### 5.1. Summary of Findings

This study comprehensively analyzed the spatiotemporal characteristics and drivers of PM_2.5_ air pollution in Shandong province from 2014 to 2023 through Geodetector and correlation analysis. PM_2.5_ concentrations exhibit distinct spatiotemporal patterns (“higher in west, lower in east” spatially and “higher in winter, lower in summer” temporally) alongside a significant long-term decreasing trend (2014–2023). Our gravity model analysis reveals a persistent concentration of PM_2.5_ pollution centroids within a stable geographic zone southwest of Zibo. Among meteorological factors, temperature-humidity coupling (q = 0.640, 0.740) and wind-pressure interactions (q = 0.576, 0.679) enhance the formation of secondary inorganic aerosols (e.g., sulfate, nitrate) and secondary organic aerosol (SOA), and induce stable atmospheric conditions unfavorable for dispersion. Among socioeconomic factors, tertiary industry production and pollution emissions are key influencing factors of PM_2.5_. PM-SO_2_ emissions synergy (q = 0.961) suggests shared combustion sources. PM-NO_x_ emissions interaction (q = 0.997) indicates transportation sector contributions. The combined effect of SO_2_ and NO_x_ emissions accounted for 99.3% of PM_2.5_ variability (q = 0.993), indicating their role as precursor pollutants. To sustainably mitigate air pollution and alleviate its associated health burdens on the Chinese population, a multi-faceted approach is crucial, encompassing stringent regulatory policies, the pursuit of carbon neutrality targets, and coordinated, regionally tailored measures. These findings provide important theoretical support for formulating targeted strategies for atmospheric pollution prevention and control.

### 5.2. Limitations and Future Research Directions

(1) Data uncertainty

PM_2.5_ data from MEE stations undergo strict quality control procedures including automatic calibration, routine maintenance, and data validity checks as per the Chinese Ministry of Ecology and Environment’s technical guidelines. Potential uncertainties may arise from instrument detection limits and occasional sensor drift.

Meteorological data from CMA stations are calibrated and validated according to the World Meteorological Organization (WMO) standards. The representativeness of point-based measurements for the entire study area is a recognized source of uncertainty.

The spatial interpolation using Ordinary Kriging introduces uncertainty, particularly in areas with sparse station coverage. The RMSE quantifies this interpolation error.

Socioeconomic data, being aggregated at the city or provincial level, may mask internal heterogeneity. Their statistical nature inherently contains reporting and estimation errors.

A key limitation is the lack of long-term PM_2.5_ chemical composition data, which prevents a more mechanistic analysis of source apportionment and aerosol transformation processes.

(2) Limits of correlation interpretation

This study identifies statistical associations rather than establishes causality. The strong correlations between socioeconomic factors and PM_2.5_, for instance, are indicative but do not prove direct cause-and-effect relationships.

(3) Impact of data quality from environmental sensors

When a sensor is directly exposed to sunlight, it heats up, causing readings to be significantly higher than the actual ambient air temperature. This is the most common source of error.

Pollutants like dust and salt can coat the sensor’s surface, impairing its moisture absorption capability. The accuracy of humidity sensors is highly dependent on temperature; an inaccurate temperature reading directly leads to an inaccurate humidity reading.

Humidity interferes with PM_2.5_ data. Fog droplets and water droplets are misinterpreted as particulate matter, leading to a severe overestimation of PM_2.5_ concentration during high-humidity conditions. Particles of different chemical compositions and colors (e.g., black carbon vs. ammonium sulfate) have different light-scattering properties, causing measurement bias. As usage time increases, the sensor’s zero point (zero drift) and sensitivity (span drift) can change, resulting in systematically high or low readings.

(4) Future Research directions

This study lacks long-term, site-specific PM_2.5_ Chemical composition data (such as black carbon, organic carbon, ions). Future research incorporating such data will provide a more detailed understanding of source apportionment and transformation mechanisms. In future studies, we will employ data from a greater number of monitoring stations and high-resolution remote sensing data, utilizing the Weather Research and Forecasting (WRF) model and the Community Multiscale Air Quality (CMAQ) model. This approach will enable a detailed analysis of the spatiotemporal characteristics of PM_2.5_ in Shandong Province and investigate the underlying mechanisms through which pollutant emissions and meteorological conditions influence PM_2.5_ levels. The findings will contribute to supporting Shandong’s synergistic achievement of its air quality goals and carbon neutrality vision.

## Figures and Tables

**Table 1:** Parameters of research methods.

Method	Key Parameters	Quality Indicators
Ordinary Kriging	Semivariogram Model: Spherical, Nugget: 0.339, Step size: 0.572	Root Mean Square Error (RMSE)
Geodetector	Interaction Detector	*q*-statistic: [0–1], *p* < 0.05

**Table 2:** Performance comparison between Kriging and Inverse Distance Weighting methods.

Interpolation Method	Root Mean Square Error (RMSE)	Slope of the Regression Function
Ordinary Kriging (OK)	6.2079	0.8342
Inverse Distance Weighting (IDW)	7.4733	0.4779

## Data Availability

The raw data supporting the conclusions of this article will be made available by the authors on request.
